# FAP-Anchored Retinoic Acid Nanoparticles for Stromal Reprogramming and Enhanced Intratumoral Oxaliplatin Delivery in Fibrotic Colorectal Tumours

**DOI:** 10.3390/bios16040189

**Published:** 2026-03-25

**Authors:** Haixia Wang, Can Xu, Ling Xie, Xiaohe Chu, Xinyu Liu, Peng Wang

**Affiliations:** 1 Collaborative Innovation Center of Yangtze River Delta Region Green Pharmaceuticals, Zhejiang University of Technology, Hangzhou 310014, China; wanghaixialzy0531@163.com (H.W.);; 2 College of Pharmaceutical Science, Zhejiang University of Technology, Hangzhou 310014, China; 3 Hangzhou Institute of Medicine (HIM), Chinese Academy of Sciences, Hangzhou 310022, China

**Keywords:** colorectal cancer, fibroblast activation protein, retinoic acid, cancer-associated fibroblast, extracellular matrix

## Abstract

In colorectal cancer (CRC), cancer-associated fibroblasts (CAFs) and the fibrotic stroma generate form a dense stromal barrier that restricts the intratumoural exposure and spatial distribution of oxaliplatin. To enable local stromal remodelling of this pathological stromal compartment, we selected fibroblast activation protein (FAP) as a stromal target and co-assembled two amphiphilic conjugates, oncoFAP and retinoic acid (RA), into an FAP-directed RA nanoformulation termed LRA^FAP^. LRA^FAP^ exhibited a uniform size distribution (107.1 ± 5.8 nm), remained stable for at least 7 d at 37 °C in PBS or serum-containing PBS, and showed accelerated esterase-responsive release. In a TGF-β-induced CAF-like model, LRA^FAP^ markedly suppressed the expression of CAF activation-associated markers, reducing Fap and Acta2 mRNA levels by approximately 70% and 60%, respectively. In vivo, LRA^FAP^ showed enhanced accumulation in CAF-enriched tumours and an increase in intratumoural oxaliplatin levels of approximately 2.5-fold relative to oxaliplatin alone. LRA^FAP^ also reduced collagen deposition and CAF activation markers, and enhanced the antitumour efficacy of oxaliplatin while maintaining good tolerability. Collectively, these findings indicate that LRA^FAP^ promotes local stromal remodelling and improves intratumoural oxaliplatin exposure, thereby enhancing the efficacy of oxaliplatin-based chemotherapy in CRC.

## 1. Introduction

CRC remains a leading cause of cancer-related morbidity and mortality worldwide and exhibits substantial biological heterogeneity [1]. In localised disease, curative-intent surgery combined with perioperative oxaliplatin-based chemotherapy, including FOLFOX or CAPOX, can improve survival outcomes [2,3]. However, a considerable proportion of patients present with locally advanced or metastatic disease and consequently have a markedly poorer prognosis [3]. In advanced or metastatic CRC, systemic treatment is typically based on a fluoropyrimidine–oxaliplatin backbone and may be combined with anti-angiogenic agents or molecularly guided targeted therapies [4]. Nevertheless, disease progression during first- or second-line treatment remains common [4,5]. Meanwhile, cumulative dose-limiting toxicities, most notably oxaliplatin-induced peripheral neuropathy, frequently necessitate dose reduction, delay, or discontinuation, thereby limiting further escalation of systemic treatment intensity [6]. Accordingly, under current standards of care, improving intratumoural drug exposure, rather than simply increasing the systemic dose, has become an important therapeutic objective in CRC [5].

Increasing evidence suggests that the limited efficacy of chemotherapy in CRC cannot be explained solely by tumour cell-intrinsic resistance, but is also shaped by a fibrotic tumour microenvironment characterised by desmoplastic stromal remodelling [7]. CRC is not merely a disease of malignant epithelial cells; rather, it develops within a dense stromal compartment comprising CAFs, tumour-associated immune cells, and abundant extracellular matrix (ECM) [8]. CAF-driven stromal remodelling promotes excessive ECM deposition and crosslinking, leading to increased solid stress and elevated interstitial fluid pressure (IFP). These changes compress microvessels and impair perfusion, thereby restricting intratumoural drug delivery and generating marked spatial heterogeneity in drug distribution. As a result, even when plasma exposure is adequate, therapeutically effective drug concentrations may not be achieved throughout the tumour tissue [5,9]. Beyond this physical barrier, persistently activated CAFs secrete cytokines such as TGF-β and IL-6, which reinforce pro-tumour signalling and contribute to a therapy-tolerant microenvironment [10,11,12]. Together, impaired drug delivery and microenvironment-driven therapeutic tolerance constitute a major barrier to effective chemotherapy in CRC.

To address this dual barrier, stromal targeting has emerged as an active area of investigation. Among stromal targets, FAP has attracted particular interest because of its high expression in pro-fibrotic CAFs. In preclinical models of multiple solid tumours, interventions targeting key stromal pathways or modulating tumour mechanical properties have been shown to attenuate ECM- and hyaluronan-associated physical barriers, improve perfusion, and enhance drug delivery and therapeutic efficacy [13,14,15]. Likewise, antibody-, ligand-, and conjugate-based approaches directed against CAF-associated targets, including FAP and PDGFR, have been explored to enhance stromal accumulation and enable local stromal remodelling [16,17]. However, clinical translation remains challenging. First, CAFs exhibit marked lineage and functional heterogeneity, and broad suppression or depletion may trigger compensatory stromal remodelling, thereby limiting the therapeutic window [18]. Second, systemic anti-fibrotic or pathway-targeted interventions are often constrained by safety considerations, while sustained local drug exposure within dense stroma remains difficult to achieve [19]. Third, the poor accessibility of fibrotic stroma, together with clearance of nanodelivery systems by the mononuclear phagocyte system, makes efficient stromal access and therapeutically effective local drug concentrations difficult to attain [20,21]. These considerations highlight the need for a delivery strategy in CRC that preferentially accumulates in FAP^+^ CAF-enriched regions and enables local stromal remodelling, thereby weakening the fibrotic barrier and increasing intratumoural oxaliplatin exposure without increasing systemic toxicity.

In the present study, we targeted the CAF-driven fibrotic microenvironment as a major therapeutic barrier in CRC and selected FAP as a stromal target to improve intratumoural exposure to standard chemotherapy. Because RA can modulate fibroblast activation and suppress collagen-rich matrix deposition through RAR/RXR-mediated transcriptional control [22,23], we used RA as a stromal reprogramming payload and developed a FAP-targeted self-assembling nanosystem, LRA^FAP^, for preferential accumulation and local RA delivery within FAP^+^ CAF-enriched stroma. In this context, we demonstrate that local stromal remodelling improves intratumoural oxaliplatin exposure and thereby enhances the antitumour efficacy of oxaliplatin-based chemotherapy without substantially increasing systemic toxicity.

## 2. Materials and Methods

### 2.1. Chemicals and Reagents

RA, oxaliplatin, DiR, the Cell Counting Kit-8 (CCK-8), and recombinant TGF-β1 protein were purchased from MedChemExpress (Monmouth Junction, NJ, USA). All other chemicals and solvents were obtained from J&K Chemicals (Shanghai, China) and Sigma-Aldrich (St. Louis, MO, USA). The Annexin V–FITC (fluorescein isothiocyanate) apoptosis detection kit was purchased from Beyotime Biotechnology (Shanghai, China).

### 2.2. Compound Synthesis

Two compounds, namely RA (linoleyl alcohol) conjugates and oncoFAP (linoleyl alcohol) conjugates, were synthesised and characterised. Their structures were confirmed by ^1^H nuclear magnetic resonance (^1^H NMR) spectroscopy using a Bruker 400 MHz spectrometer (Bruker, Billerica, MA, USA). The synthetic routes and characterisation data are provided below.

RA (100 mg, 0.34 mmol, 1.0 equiv.) and linoleyl alcohol (114.4 mg, 0.43 mmol, 1.3 equiv.) were dissolved in anhydrous dichloromethane (3 mL). 4-(Dimethylamino)pyridine (DMAP; 52.5 mg, 0.43 mmol, 1.3 equiv.) and 1-ethyl-3-(3-dimethylaminopropyl)carbodiimide (EDC; 66.7 mg, 0.43 mmol, 1.3 equiv.) were then added, and the reaction mixture was stirred at 40 °C overnight. Upon completion, the mixture was extracted with dichloromethane. The combined organic layers were washed sequentially with 5% (*w*/*v*) aqueous citric acid, saturated aqueous sodium bicarbonate, and saturated brine, then dried over anhydrous sodium sulphate. Purification by silica gel column chromatography (dichloromethane/methanol as the eluent) produced the desired product as a yellow solid.

RA–linoleyl alcohol conjugates: ^1^H NMR (400 MHz, Chloroform-*d*) δ 6.99 (dd, *J* = 15.1, 11.4 Hz, 1H), 6.33–6.10 (m, 4H), 5.36 (qd, *J* = 11.0, 5.2 Hz, 5H), 4.10 (t, *J* = 6.7 Hz, 2H), 2.78 (t, *J* = 6.5 Hz, 2H), 2.36 (d, *J* = 1.0 Hz, 3H), 2.08–1.98 (m, 10H), 1.72 (s, 3H), 1.62 (ddd, *J* = 14.7, 8.2, 3.8 Hz, 4H), 1.39–1.26 (m, 21H), 1.03 (s, 6H), 0.89 (t, *J* = 6.8 Hz, 4H).

OncoFAP (100 mg, 0.22 mmol, 1.0 equiv.) and linoleyl alcohol (75 mg, 0.28 mmol, 1.3 equiv.) were dissolved in anhydrous N,N-dimethylformamide (DMF, 3 mL). DMAP (34.2 mg, 0.28 mmol, 1.3 equiv.) and EDC (43.9 mg, 0.28 mmol, 1.3 equiv.) were then added, and the reaction mixture was stirred at 40 °C overnight. Upon completion, the mixture was extracted with dichloromethane. The combined organic layers were washed sequentially with 5% (*w*/*v*) aqueous citric acid, saturated aqueous sodium bicarbonate, and saturated aqueous sodium chloride (brine), dried over anhydrous sodium sulphate, and concentrated under reduced pressure. The crude product was purified by silica gel column chromatography using dichloromethane/methanol as the eluent to produce the product as a pale-yellow solid.

OncoFAP–linoleyl alcohol conjugates: ^1^H NMR (400 MHz, DMSO-*d*_6_) δ 10.25 (d, *J* = 6.4 Hz, 1H), 9.17 (t, *J* = 6.0 Hz, 1H), 9.02 (dd, *J* = 4.3, 2.3 Hz, 1H), 8.64 (dt, *J* = 7.9, 1.6 Hz, 1H), 8.01–7.93 (m, 2H), 7.69–7.58 (m, 2H), 5.38–5.26 (m, 3H), 5.19 (dd, *J* = 9.3, 2.6 Hz, 1H), 4.38–4.09 (m, 4H), 4.01 (t, *J* = 6.5 Hz, 2H), 2.89 (s, 4H), 2.73 (s, 4H), 2.00 (dt, *J* = 11.4, 5.5 Hz, 4H), 1.54 (p, *J* = 6.6 Hz, 2H), 1.28–1.18 (m, 16H), 0.89–0.81 (m, 3H). The synthetic routes are shown in Supplementary Figure S1.

### 2.3. Preparation of FAP-Targeted Prodrug Nanoassemblies

RA (linoleyl alcohol) conjugates and oncoFAP (linoleyl alcohol) conjugates were co-assembled into nanoassemblies using the nanoprecipitation method. Briefly, the two prodrug conjugates were dissolved in dimethyl sulfoxide (DMSO; 1 mL) at a molar ratio of 5:1, and the solution was slowly added dropwise to deionised water (9 mL) under sonication to form co-assembled nanoparticles. Residual DMSO was subsequently removed by dialysis against deionised water. The final drug concentration was determined by UV–vis spectrophotometry (UV-2600, Shimadzu, Kyoto, Japan).

### 2.4. Characterisation and Stability Assessment of the LRA^FAP^ Nanoassemblies

The hydrodynamic diameter and polydispersity index (PDI) of LRA^FAP^ were determined by dynamic light scattering (DLS) using a Zetasizer Nano ZS90 (Malvern Instruments, Malvern, UK). Particle morphology was examined by transmission electron microscopy (TEM) using a JEM-1400 microscope (JEOL, Tokyo, Japan) operated at 120 kV. Briefly, samples were deposited onto carbon-coated 300-mesh copper grids. After approximately 2 min, excess liquid was wicked off and the grids were air-dried. The dried specimens were then negatively stained with 2% (*w*/*v*) aqueous uranyl acetate and imaged using TEM at an accelerating voltage of 120 kV. For the stability study, changes in particle size and PDI were monitored by DLS over 7 days.

### 2.5. In Vitro Release Study of LRA^FAP^

To evaluate the cumulative in vitro release of LRA^FAP^, samples were gently agitated at 37 °C in different release media. Briefly, preformed LRA^FAP^ nanoassemblies (equivalent to 0.5 mg mL^−1^ RA) were loaded into dialysis bags and immersed in the respective media. The nanoassembly suspension was maintained at 37 °C under gentle shaking. At predetermined time points, 1 mL of the release medium was withdrawn and immediately replaced with an equal volume of fresh medium. The collected samples were analysed by UV–vis spectrophotometry to determine the concentration of RA, and the cumulative drug release was calculated accordingly.

### 2.6. Cell Lines and Cell Culture

The murine colon carcinoma cell line MC38 and the murine embryonic fibroblast cell line NIH/3T3 were obtained from the Cell Bank of the Chinese Academy of Sciences (Shanghai, China). MC38 and NIH/3T3 cells were cultured in Dulbecco’s modified Eagle’s medium (DMEM) supplemented with 10% foetal bovine serum (FBS), penicillin (100 U mL^−1^) and streptomycin (100 μg mL^−1^). Cells were maintained at 37 °C in a humidified incubator with 5% CO_2_. To generate activated fibroblasts with a cancer-associated fibroblast-like phenotype, NIH/3T3 cells were serum-starved overnight in FBS-free DMEM and then treated with recombinant human transforming growth factor-β1 (TGF-β1; 20 ng mL^−1^) for 48 h.

### 2.7. In Vitro Cytotoxicity Assay

The Cell Counting Kit-8 (CCK-8) assay was used to evaluate the in vitro cytotoxicity of the indicated compounds in different cell lines. Briefly, MC38 or NIH/3T3 cells were seeded into sterile 96-well plates at a density of 2 × 10^3^ cells per well and incubated overnight. The medium was then replaced with fresh culture medium containing the compounds at various concentrations, followed by further incubation for 72 h. After treatment, cell viability was assessed using the CCK-8 assay, and absorbance was measured at 450 nm using a microplate reader (BioTek Synergy H1, BioTek Instruments, Winooski, VT, USA).

### 2.8. Flow Cytometric Analysis of Apoptosis

Apoptosis was evaluated by flow cytometry using an Annexin V–FITC/propidium iodide (PI) apoptosis detection kit, followed by analysis on an Attune NxT flow cytometer (Thermo Fisher Scientific, Waltham, MA, USA). MC38 or NIH/3T3 cells were seeded into 6-well plates at a density of 8 × 10^4^ cells per well and incubated overnight at 37 °C. Cells were then treated with the compounds at a range of concentrations and incubated for 48 h at 37 °C. At the end of the incubation period, cells were harvested, washed twice with PBS, and resuspended in binding buffer. Annexin V–FITC and PI were added to the cell suspension according to the manufacturer’s instructions. After washing, apoptotic cells were quantified and analysed by flow cytometry.

### 2.9. Ethics Declaration

The study was approved by the Animal Ethics Committee of the Hangzhou Institute of Medicine, Chinese Academy of Sciences (protocol code: AP2026-01-0719; date: 30 July 2025). The procedures complied with international guidelines for the ethical use of laboratory animals.

### 2.10. Establishment of the Tumour-Bearing Mouse Model

Female C57BL/6J mice were selected for tumour model establishment. To generate a fibrotic MC38 model, a mixture of MC38 cells and activated fibroblasts was prepared at a 2:1 ratio and implanted subcutaneously into the lower abdominal region of the mice. For the nanoassembly tumour-targeting study, the fibrotic MC38 model was established by inoculating the MC38/activated fibroblast mixture subcutaneously into the right flank, while an equivalent number of MC38 cells alone was inoculated subcutaneously into the left flank.

### 2.11. In Vivo Biodistribution and Tumour-Targeting Evaluation of the Nanoassemblies

DiR was loaded into LRA^FAP^ to investigate in vivo biodistribution and tumour-targeting performance. Female C57BL/6J mice bearing bilateral subcutaneous tumours (established as described above) were intravenously injected with DiR-labelled nanoassemblies (LRA^FAP^@DiR). At 2, 4, and 12 h post-injection, mice were anaesthetised and imaged using an in vivo optical imaging system. At 12 h post-injection, all mice were euthanised, and major organs (heart, liver, spleen, lung, and kidney) as well as the intestine were harvested for ex vivo imaging and quantitative analysis using the IVIS imaging system (PerkinElmer, Waltham, MA, USA). To assess intratumoural accumulation, tumour tissues were prepared as cryosections and counterstained with DAPI. The frozen sections were imaged using confocal laser scanning microscopy (CLSM; FV3000, Olympus, Tokyo, Japan).

### 2.12. Quantification of Intratumoural Oxaliplatin Content

To evaluate the effect of LRA^FAP^ pretreatment on intratumoural oxaliplatin content, an MC38/CAF mixed subcutaneous tumour model was established. MC38 cells were mixed with CAFs and inoculated subcutaneously into C57BL/6 mice. When the tumour volume reached approximately 80 mm^3^, the mice were randomly divided into two groups. The experimental group received LRA^FAP^ pretreatment, whereas the control group received an equal volume of normal saline; both groups were subsequently administered oxaliplatin via tail-vein injection. Mice were sacrificed 24 h after a single dose of oxaliplatin, and the tumours were rapidly excised and weighed. Collected tumour tissues were homogenised in pre-cooled homogenisation buffer, followed by protein precipitation. After centrifugation, the supernatants were collected and analyzed by HPLC on an LC-20 system (Shimadzu Corporation, Kyoto, Japan) Oxaliplatin content was determined by HPLC using a Shim-pack GIST C18 reversed-phase column (Shimadzu Corporation, Kyoto, Japan), with the column temperature set at 10 °C, an injection volume of 100 μL, ultraviolet detection at 254 nm, and a flow rate of 1.0 mL/min. Quantification of oxaliplatin in the tumour samples was performed using a standard curve generated from oxaliplatin standards, and the measured values were normalised to tumour weight. The results were expressed as μg/g tumour tissue. The dosing regimens and routes of administration for LRA^FAP^ and oxaliplatin were as described in the Section 2.13.

### 2.13. In Vivo Antitumour Activity in Tumour Models

Female C57BL/6J mice were subcutaneously inoculated with a mixed cell suspension containing murine colon carcinoma MC38 cells (1 × 10^6^) and activated fibroblasts (5 × 10^5^). When tumours reached approximately 80 mm^3^, the mice were randomly assigned to five groups and the following treatments were initiated: saline; RA (10 mg kg^−1^, oral gavage); LRA^FAP^ (5 mg kg^−1^, intravenous injection); oxaliplatin (15 mg kg^−1^, intravenous injection); or LRA^FAP^ (5 mg kg^−1^, intravenous injection) in combination with oxaliplatin. Tumour volume and body weight were measured and recorded every 2 days. On day 8, mice from each group were euthanised; tumours were fixed in formaldehyde solution for Masson’s trichrome staining or immunofluorescence staining. Blood samples were also collected for biochemical analysis. Tumour volume was estimated using the following formula: length × width^2^ × 0.5.

### 2.14. Statistical Analysis

All statistical analyses were performed using GraphPad Prism 8.0 (GraphPad Software, Inc., La Jolla, CA, USA). Quantitative data are presented as the mean ± standard deviation (SD). Statistical significance among multiple groups was assessed using one-way or two-way analysis of variance (ANOVA), as appropriate, followed by Tukey’s multiple-comparisons test, as indicated in the figure legends. Statistical significance was set at *p* < 0.05 (*p* < 0.05, *p* < 0.01, *p* < 0.001, *p* < 0.0001); “ns” denotes not significant.

## 3. Results

### 3.1. Preparation of Co-Assembled LRA^FAP^ Nanoparticles and Their In Vitro Release Behaviour

To construct the LRA^FAP^ nanoformulation, we first synthesised two amphiphilic conjugates, oncoFAP (linoleyl alcohol) and retinoic acid (linoleyl alcohol), and confirmed their structures (see Section 2). The design was intended to enable targeted delivery within the tumour microenvironment by recognising fibroblast FAP expressed on the surface of CAFs. Prior studies have shown that such prodrug constructs can possess sufficient amphiphilicity to self-assemble in aqueous media via nanoprecipitation, yielding water-dispersible nanoparticles [24]. Moreover, owing to their inherent self-assembly capability, the formulation can be prepared without additional excipients, thereby potentially reducing excipient-related risks.

**Figure 1:**
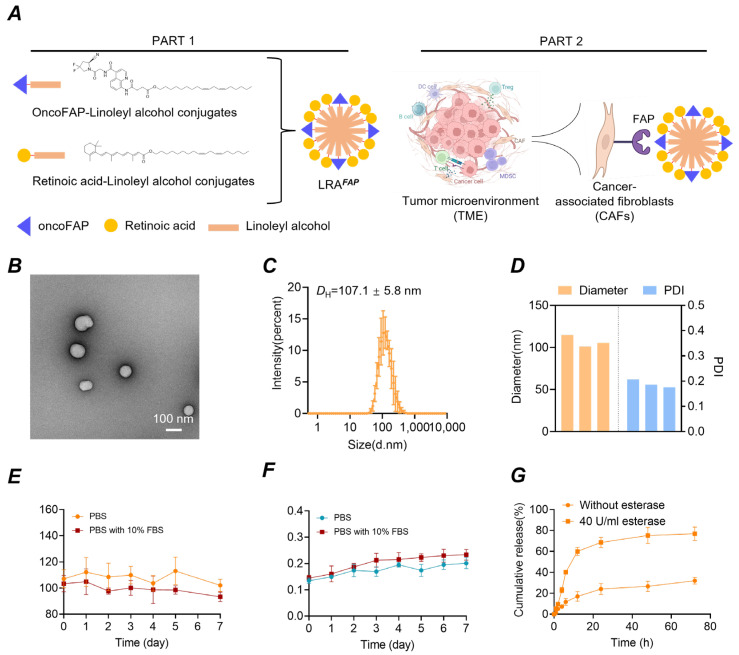
Fabrication, morphology, dispersity, stability and esterase-responsive release of LRA^FAP^ nanoparticles. (**A**) Amphiphilic conjugates oncoFAP–linoleyl alcohol and retinoic acid–linoleyl alcohol co-assemble to form LRA^FAP^, which was designed to enable target-directed delivery by recognising FAP on the surface of CAFs. (**B**) TEM reveals near-spherical particles with good dispersity (scale bar: 100 nm). (**C**) The intensity-weighted DLS size distribution is unimodal, with a Z-average of 107.1 ± 5.8 nm. (**D**) Comparison of hydrodynamic diameter (left axis, nm) and PDI (right axis) across independent batches indicates good inter-batch consistency. (**E**,**F**) Nanoparticles stored in PBS (pH 7.4) or PBS (pH 7.4) containing 10% FBS at 37 °C for 7 days show no appreciable changes in particle size or PDI over time. (**G**) At 37 °C in the presence of esterase (40 U/mL), cumulative release is markedly faster than the no-esterase control.

We found that these two conjugates co-assembled in aqueous conditions to form LRA^FAP^ nanoparticles with a uniform morphology and size (Figure 1A). Notably, no visible precipitation was observed after dialysis to further remove DMSO, indicating that the colloidal stability of the assemblies was maintained after organic-solvent removal. Transmission electron microscopy (TEM) revealed well-dispersed, quasi-spherical particles (Figure 1B; scale bar, 100 nm). DLS showed a unimodal size distribution (Figure 1C), with a mean hydrodynamic diameter of 107.1 ± 5.8 nm. The hydrodynamic diameter and polydispersity index (PDI) were comparable across independent batches (Figure 1D), demonstrating good uniformity and batch-to-batch reproducibility. In addition, stability studies indicated that, over one week in PBS or serum-containing PBS at 37 °C, the nanoparticle diameter remained unchanged and no precipitation was detected (Figure 1E,F). Collectively, these data confirm the successful construction of the nanoassemblies, with a tuneable drug ratio to facilitate optimisation of potential combinatorial effects within the scaffold. The improved aqueous dispersibility also provides a formulation basis for subsequent preclinical evaluation via intravenous administration. Given the ester functionalities incorporated into the prodrug design, we further assessed esterase-responsive release. In the absence of esterase, release proceeded relatively slowly; by contrast, in the presence of esterase (40 U/mL), the cumulative release rate increased markedly (Figure 1G), supporting an esterase-triggered drug-release behaviour for this system.

### 3.2. In Vitro Safety, CAF Modulation, Tumour Targeting and Intratumoural Oxaliplatin Accumulation of LRA^FAP^

**Figure 2:**
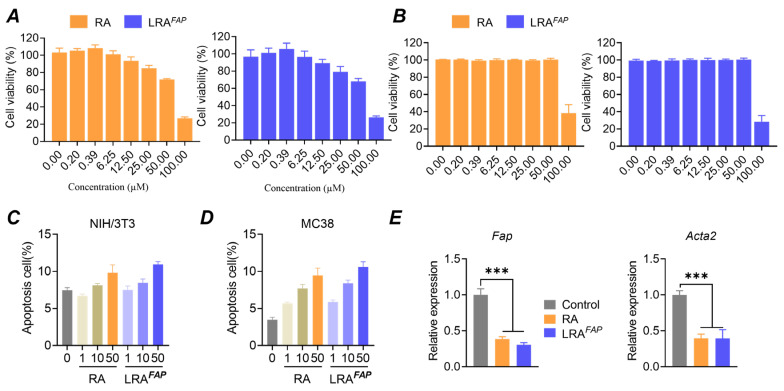
In vitro safety profile of LRA^FAP^ and its modulation of CAF-associated markers. (**A**,**B**) Cell viability of NIH/3T3 fibroblasts (**A**) and MC38 tumour cells (**B**) following treatment with free RA or LRA^FAP^ at the indicated concentrations. (**C**,**D**) Apoptosis analysis (early/late) of NIH/3T3 cells (**C**) and MC38 cells (**D**) after exposure to RA or LRA^FAP^ at the indicated doses. (**E**) In a TGF-β-induced CAF-like model, effects of free RA and LRA^FAP^ on the relative expression of CAF activation-related genes Fap and Acta2 (encodes α-SMA). *** *p* < 0.001.

Across the 0–100 μM dose range, RA and LRA^FAP^ exerted minimal effects on overall cell viability in NIH/3T3 and MC38 cells. At concentrations ≤50 μM, viability remained high in both cell lines, showing only a modest dose-dependent decline (Figure 2A,B), whereas at 100 μM, both treatments caused a pronounced reduction in viability. Consistent with these findings, Annexin V/PI analysis indicated that, across the tested doses, the proportions of early and late apoptotic NIH/3T3 and MC38 cells remained low, with only a slight increase at higher concentrations and no evident accumulation of apoptosis (Figure 2C,D).

In a TGF-β-induced CAF-like NIH/3T3 model, qPCR analysis showed that both RA and LRA^FAP^ significantly downregulated the transcript levels of CAF activation-associated genes, with LRA^FAP^ producing a greater magnitude of suppression, reducing their expression by approximately 70% and 60%, respectively (Figure 2E, *** *p* < 0.001). On the basis of these dose–toxicity and phenotype-modulation results, 10 μM was selected as the working concentration for subsequent in vitro functional assays to evaluate stromal reprogramming under conditions that did not materially affect cell viability or apoptosis. Validation of TGF-β induction and the CAF-like phenotype is provided in Supplementary Figure S2.

**Figure 3:**
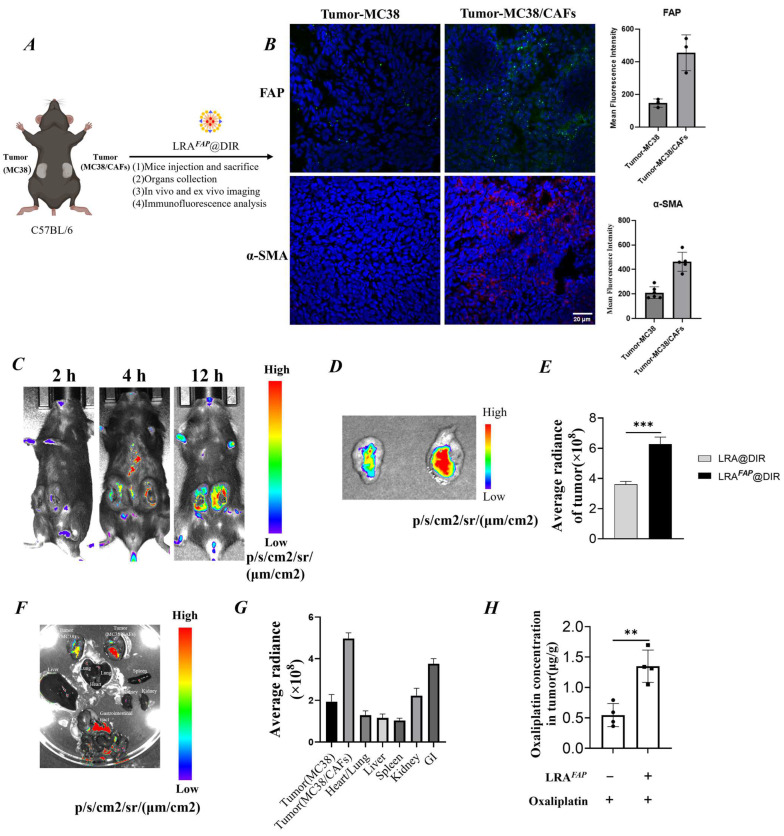
In vivo targeting and biodistribution of LRA^FAP^@DiR in CAF-enriched (MC38/CAF) tumours. (**A**) Schematic of the bilateral subcutaneous tumour model and the dosing/imaging schedule. (**B**) Immunofluorescence staining of tumour sections (FAP, α-SMA, DAPI) confirming establishment of a CAF-enriched model. (**C**) Near-infrared in vivo imaging (2, 4 and 12 h) showing time-dependent accumulation of LRA^FAP^@DiR in CAF-enriched (MC38/CAF) tumours. (**D**,**E**) Ex vivo tumour imaging and ROI quantification: the LRA^FAP^@DiR signal is higher than that of the control LRA@DiR. (**F**,**G**) Ex vivo near-infrared fluorescence biodistribution of LRA^FAP^@DiR in a bilateral subcutaneous tumour model comprising MC38- and CAF-enriched MC38/CAF tumours: (**F**) representative ex vivo images of excised tumours and major organs; (**G**) ROI-based quantification of fluorescence signals in tumours and organs. The higher signal observed in the CAF-enriched (MC38/CAF) tumour is indicative of preferential accumulation within the FAP^+^ stromal compartment. (**H**) Intratumoural oxaliplatin concentration in the MC38/CAF tumour model with or without LRA^FAP^ pretreatment. ** *p* < 0.01, *** *p* < 0.001.

We established a bilateral subcutaneous tumour model in the same C57BL/6 mouse (left: MC38; right: MC38/CAFs), followed by intravenous administration of LRA^FAP^@DiR, and performed in vivo/ex vivo imaging together with histological validation (Figure 3A). First, triple immunofluorescence staining of tumour sections confirmed that FAP and α-SMA were markedly upregulated in the CAF-enriched tumour, indicating successful model establishment (Figure 3B). Time-course in vivo near-infrared imaging subsequently showed that LRA^FAP^@DiR accumulated in the CAF-enriched tumour in a time-dependent manner, with the signal progressively increasing and reaching a maximum within the observation window. By contrast, fluorescence in the contralateral MC38 tumour and non-tumour regions remained low, whereas the control formulation LRA@DiR exhibited an overall lower tumour-to-background ratio (Figure 3C). Consistent with the in vivo observations, ex vivo tumour imaging and ROI quantification showed that LRA^FAP^@DiR exhibited a significantly higher mean radiant efficiency than LRA@DiR, representing an approximately 1.7-fold increase (Figure 3D,E, *p* < 0.001). Notably, separate analysis of the bilateral tumours showed that LRA@DiR displayed only limited differences between MC38 and MC38/CAF tumours, whereas LRA^FAP^@DiR exhibited markedly greater accumulation in the MC38/CAF tumours (Supplementary Figure S3). Organ biodistribution imaging further indicated enhanced tumour accumulation of LRA^FAP^@DiR, with a stronger signal in the CAF-enriched MC38/CAF tumours, consistent with preferential localisation in the FAP/CAF-associated stromal compartment (Figure 3F,G).To further determine whether the above imaging findings were reflected in higher intratumoural drug levels, we additionally quantified the oxaliplatin content in tumour tissues using an independently established subcutaneous MC38/CAF model. The results showed that, compared with oxaliplatin alone, pretreatment with LRA^FAP^ significantly increased intratumoural oxaliplatin concentrations to approximately 2.5-fold that of the control group (Figure 3H, *p* < 0.01). These findings indicate that LRA^FAP^ not only shows preferential accumulation in CAF-enriched tumours, but may also enhance the intratumoural exposure of subsequently administered oxaliplatin through remodelling of the tumour stroma.

### 3.3. In Vivo Antitumour Efficacy, Safety and Stromal Remodelling Effects of LRA^FAP^

**Figure 4:**
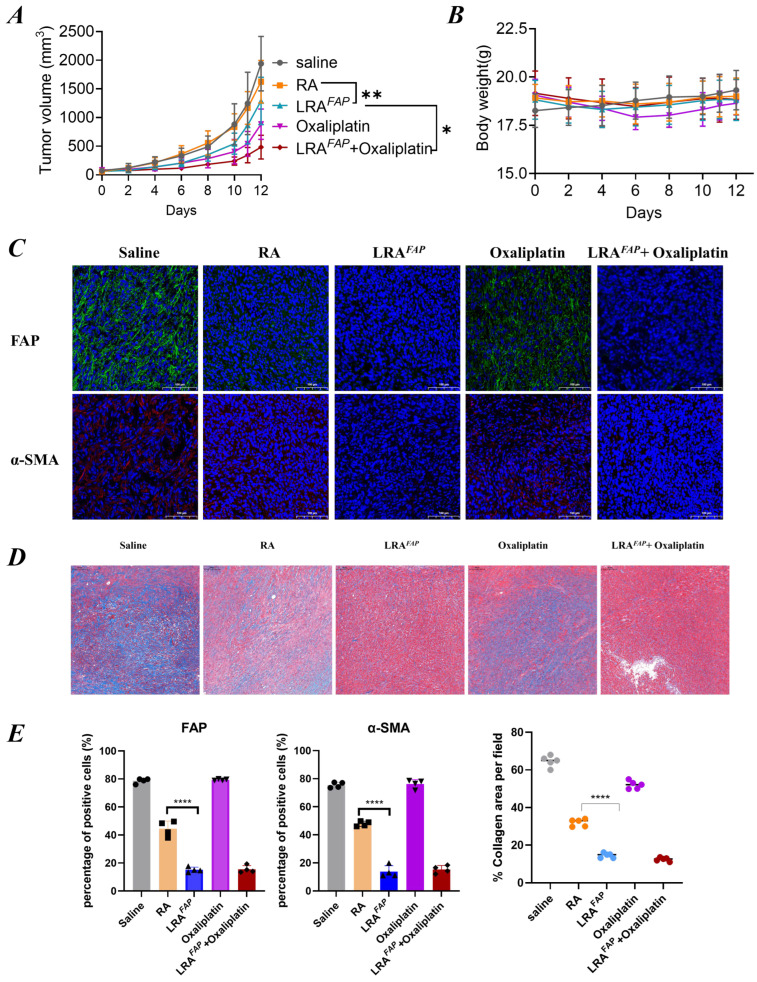
Antitumour efficacy, safety and tumour stroma remodelling in tumour-bearing mice under different treatment regimens. (**A**) Tumour volume growth curves over time (mm^3^). (**B**) Body weight changes over time, used to assess overall tolerability. (**C**) Immunofluorescence staining of tumour sections: FAP (green) and α-SMA (red), with nuclei counterstained with DAPI (blue). (**D**) Masson’s trichrome staining to assess collagen deposition in tumours, with representative images shown. (**E**) Quantification of the proportions of FAP^+^ and α-SMA^+^ cells, together with the percentage collagen area per field (% collagen area per field). Treatment groups included saline, RA, LRA^FAP^, oxaliplatin, and LRA^FAP^ + oxaliplatin. * *p* < 0.05, ** *p* < 0.01, **** *p* < 0.0001.

On the basis that LRA^FAP^ was shown to increase intratumoural oxaliplatin content, we next evaluated whether this stromal-targeting strategy could translate in vivo into improved antitumour efficacy together with acceptable systemic safety. To this end, therapeutic efficacy and safety were compared in tumour-bearing mice receiving normal saline, free RA, LRA^FAP^, oxaliplatin alone, or the combination of LRA^FAP^ and oxaliplatin (Figure 4). Relative to the control group, free RA produced only limited inhibition of tumour growth. In contrast, LRA^FAP^ markedly delayed tumour volume progression (Figure 4A) and resulted in a smaller percentage change in tumour volume between baseline and the study endpoint (Figure S4). Moreover, the combination of LRA^FAP^ and oxaliplatin achieved stronger tumour suppression than either monotherapy (Figure 4A), suggesting that, in this model, LRA^FAP^ can enhance the overall therapeutic benefit of platinum-based chemotherapy. With respect to systemic safety, body weight remained broadly stable across all groups during the dosing period (Figure 4B), and no treatment-associated, overt body weight loss was observed. Serum biochemistry analysis further showed that the ALT, AST and BUN levels were not significantly different from those of the control group in any of the treatment arms (Figure S5), indicating no detectable signals of hepatic or renal toxicity within the dosing regimen and observation window employed.

To further investigate microenvironmental changes that may contribute to the improved in vivo efficacy of LRA^FAP^, we performed histological assessments of tumour stroma-related parameters. Immunofluorescence staining demonstrated that, compared with saline or free RA, LRA^FAP^ more substantially reduced FAP+ and α-SMA+ signals within tumour tissues (Figure 4C). Quantitative analysis indicated a more effective suppression of CAF activation (Figure 4E left and middle). Consistently, Masson’s trichrome staining revealed decreased collagen deposition following LRA^FAP^ treatment, accompanied by a reduced percentage collagen area (Figure 4D,E (right)).

Collectively, LRA^FAP^ attenuated stromal fibrotic features characterised by reduced CAF activation and diminished collagen accumulation. These histological alterations are concordant with the observed enhancement of oxaliplatin antitumour activity in the combination regimen, supporting the notion that alleviation of stromal fibrosis may represent an important microenvironment-associated mechanism through which LRA^FAP^ improves the overall efficacy of platinum-based chemotherapy.

## 4. Discussion

In CRC characterised by marked fibrosis and CAF enrichment, stromal barriers limit local drug exposure and contribute to therapeutic heterogeneity [25,26,27]. Against this background, our findings suggest that the FAP-directed nanosystem LRA^FAP^ preferentially accumulates in CAF-enriched tumours, attenuates stromal activation, and increases intratumoural oxaliplatin levels. These changes are consistent with partial relief of stromal barriers and improved exposure to subsequently administered oxaliplatin. Notably, LRA^FAP^ monotherapy also showed measurable antitumour activity, which was further enhanced in combination with oxaliplatin while maintaining an overall favourable tolerability profile.

Given its preferential stromal distribution in CAF-enriched tumours, it is plausible that a substantial proportion of the pharmacological activity of LRA^FAP^ is exerted within the fibrotic CAF–ECM compartment. The oncoFAP moiety may contribute to preferential stromal accumulation and thereby help confine RA action to this compartment. Within this setting, RA may, at least in part, attenuate profibrotic programmes through RAR/RXR-mediated signalling, thereby limiting ECM deposition and stromal stiffening. This interpretation is consistent with our findings that LRA^FAP^ attenuated CAF activation-associated markers and reduced the collagen burden. In addition, if CAF activation is attenuated, the tumour-supportive functions of CAFs may also be diminished, which may partly explain the antitumour activity observed with LRA^FAP^ monotherapy. Notably, the constraint imposed by the CAF–ECM axis on chemotherapy efficacy is not limited to impaired molecular diffusion; collagen deposition and ECM cross-linking may also render the stroma denser and stiffer, accompanied by elevated interstitial fluid pressure and compression of the microvasculature, thereby impairing perfusion and convective transport [9,28,29,30,31]. Taken together, these findings support a predominantly stromal mechanism of action in which LRA^FAP^ may help alleviate structural and transport-related barriers associated with the CAF–ECM axis, potentially creating a microenvironment more permissive to subsequent drug delivery into tumours.

Prior work on FAP-directed stromal intervention has broadly fallen into three categories: FAP-specific molecular imaging and radioligand therapy in which target expression and spatial distribution serve as quantifiable readouts for patient stratification and therapeutic monitoring [32,33]; oncoFAP-based targeting strategies that enable localised drug release within the FAP^+^ stromal compartment via cleavable or activatable linkers [17,34]; and effector approaches, such as FAP-CAR-M, which were designed to directly eliminate FAP^+^ CAFs, reduce stromal collagen burden, and enhance responsiveness to other therapies [35]. Notably, antifibrotic intervention may be an inherently double-edged sword: on the one hand, attenuation of fibrosis facilitates drug penetration and immune-cell infiltration into tumours; on the other, in certain contexts, it may weaken the restraining, barrier-like functions of the stroma and thereby create conditions conducive to tumour invasion and metastasis [18,36]. This indicates that the relationship between CAFs, fibrosis, and tumour behaviour does not fit a simplistic depletion-centric paradigm; rather, its net effect depends upon the composition of CAF subsets, as well as the intensity and timing of the intervention. Consequently, compared with indiscriminate depletion, a more controlled strategy of stromal remodelling may better mitigate barrier effects while reducing the risks associated with excessive disruption of stromal architecture.

Against this backdrop, we position the present study as a stroma-sensitisation strategy centred on “remodelling rather than ablation”. Specifically, we use FAP-associated stroma as the point of intervention to controllably modulate stromal state within the chemotherapy time window, thereby potentially alleviating stromal-barrier constraints on the delivery of standard cytotoxic regimens. RA has the capacity to regulate fibroblast activation and the synthesis of fibrosis-associated matrix; however, free RA is limited by its poor solubility, unfavourable systemic exposure, and the risk of off-target distribution [37,38]. We therefore incorporated RA into a FAP-directed nanosystem as a stromal reprogramming component to sensitise tumours to chemotherapy, rather than as a replacement for cytotoxic treatment itself. In essence, this design is intended to remodel the stromal microenvironment to improve intratumoural exposure to subsequently administered oxaliplatin, rather than to deplete the stroma outright.

The durability, reversibility, and longer-term applicability of this strategy warrant further investigation. The present findings primarily indicate an improvement in stromal phenotype within the dosing regimen and observation window examined here; how long such changes persist after treatment cessation remains to be determined in longer-term studies. This strategy relies principally on RA–RAR/RXR-mediated transcriptional regulation and may therefore, in principle, be compatible with a reversible form of phenotypic reprogramming. Given the plasticity of CAF states and the relatively slow turnover and re-establishment of ECM density, we speculate that this approach may create a stromal-sensitising window during key chemotherapy cycles that facilitates subsequent drug penetration into tumours. Consistent with this view, intratumoural oxaliplatin levels were markedly increased following LRA^FAP^ pretreatment, suggesting that this strategy may improve stromal remodelling while also enhancing intratumoural exposure to subsequently administered oxaliplatin. Over longer time scales, its broader applicability and potential risks remain to be clarified. Excessive attenuation of fibrosis may alter stromal niche constraints that help restrain metastatic dissemination. In addition, FAP may be inducibly expressed in non-malignant tissue repair, inflammation, or fibrotic conditions, and careful evaluation of both off-tumour exposure and the therapeutic window under more intensive or prolonged dosing regimens will therefore be required. Moreover, the RA–RAR/RXR axis may influence treatment responses through effects on the immune microenvironment; nevertheless, within the current observation window, the available evidence supports a predominantly stromal interpretation, and the precise contribution of immune-related mechanisms remains to be delineated.

In summary, the present study highlights the important role of tumour stroma in shaping intratumoural exposure to oxaliplatin-based chemotherapy and supports a stromal-sensitising strategy centred on “remodelling rather than depletion”. By preferentially accumulating in a high-FAP stromal compartment and gently modulating the CAF–ECM axis, this approach may enhance intratumoural exposure to subsequently administered oxaliplatin, thereby contributing to improved therapeutic efficacy. Importantly, CAFs do not represent a uniform population, but rather a heterogeneous set of stromal cell states with distinct lineage and functional characteristics. Although FAP is widely used as a CAF-associated marker, it is more strongly associated with subpopulations involved in fibrotic activity and does not necessarily capture the full spectrum of CAF states. Accordingly, the present findings indicate preferential accumulation within a high-FAP, fibrosis-associated stromal compartment under the specific model and observation window examined here. The robustness and broader applicability of this interpretation will need to be tested in models of greater clinical fidelity. Overall, this work provides a testable mechanistic framework for understanding how stromal remodelling may translate into therapeutic benefits and offers a conceptual basis for therapeutic intervention in CAF-enriched, highly fibrotic CRC.

## 5. Conclusions

This study addressed the stromal drug-delivery barrier in CRC driven by CAF enrichment and fibrosis, and established a stromal sensitisation strategy centred on functional reprogramming. We developed an FAP-targeted retinoic-acid nanoformulation, LRA^FAP^, which exhibited stable nanoscale characteristics, good short-term colloidal stability, and esterase-responsive release behaviour, thereby supporting the intended sequence of stroma-preferential accumulation, localised release, and phenotypic modulation. The in vitro and in vivo findings showed that LRA^FAP^ preferentially targeted FAP-associated tumour stroma, reduced CAF activation-associated markers, and significantly increased intratumoural oxaliplatin levels, supporting the interpretation that stromal remodelling enhanced subsequent intratumoural exposure to chemotherapy. Overall, this study highlights the important role of tumour stroma in shaping effective intratumoural exposure to standard therapies and establishes a stromal sensitisation paradigm based on remodelling rather than depletion. This strategy offers a translationally relevant approach to alleviating delivery constraints in fibrotic tumours and enhancing the efficacy of platinum-based therapy in CAF-enriched, highly fibrotic CRC.

## Data Availability

The original contributions presented in this study are included in the article/Supplementary Materials. Further inquiries can be directed to the corresponding authors.

## Supplementary Materials

The following supporting information can be downloaded at: https://www.mdpi.com/article/10.3390/bios16040189/s1, Supplementary Figure S1: Synthetic routes for RA (linoleyl alcohol) and oncoFAP (linoleyl alcohol) conjugates. Supplementary Figure S2: Validation of a TGF-β-induced CAF-like NIH/3T3 model. Supplementary Figure S3: Quantitative comparison of tumour accumulation between MC38 and MC38/CAF tumours in the bilateral subcutaneous model. Supplementary Figure S4: Percentage change in tumour volume between baseline and study endpoint. Supplementary Figure S5: Serum biochemistry indices for hepatic and renal toxicity evaluations.
