# Supplementary material for: FAP-Anchored Retinoic Acid Nanoparticles for Stromal Reprogramming and Enhanced Intratumoral Oxaliplatin Delivery in Fibrotic Colorectal Tumours
Source: Biosensors (Basel). 2026 Mar 25;16(4):189. doi: 10.3390/bios16040189 (PMC13115307; doi:10.3390/bios16040189)
Supplement: Supplementary file 1 [file biosensors-16-00189-s001.zip › biosensors-4134233-supplementary.pdf]

Figure S1

A Retinoic acid-Linoleyl alcohol conjugates

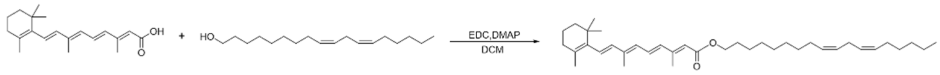

B OncoFAP-Linoleyl alcohol conjugates

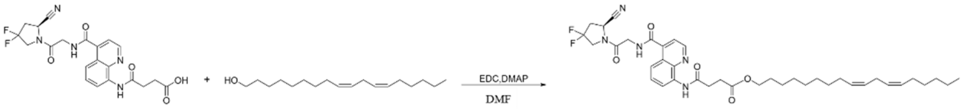

Figure S2

A

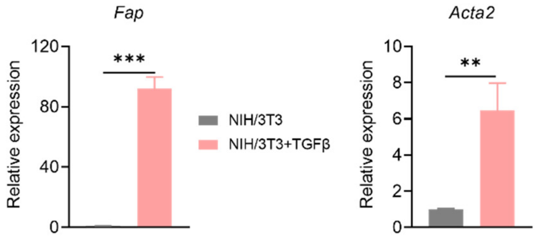

Figure S3

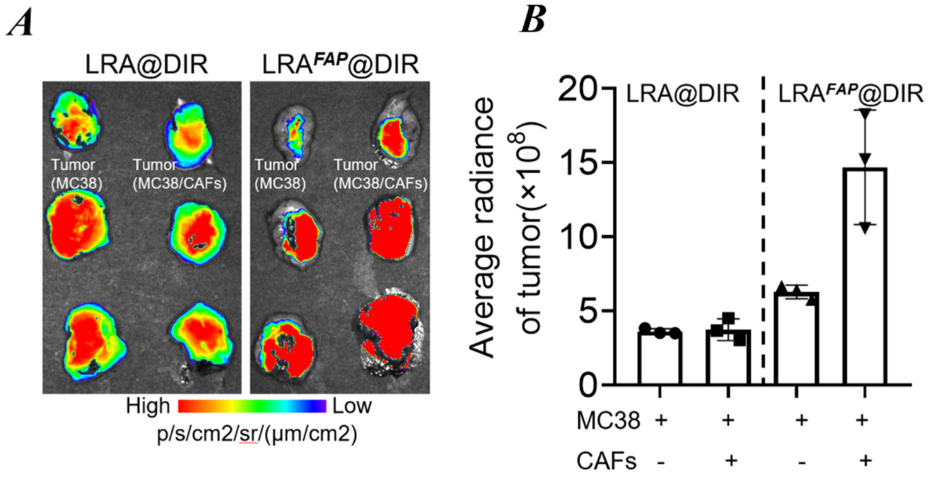

Figure S4

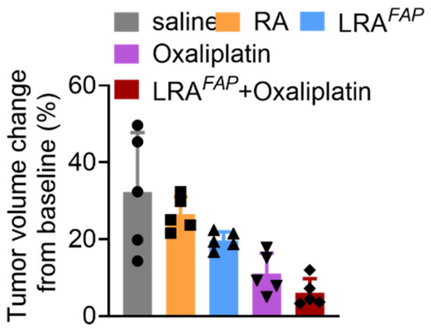

Figure S5

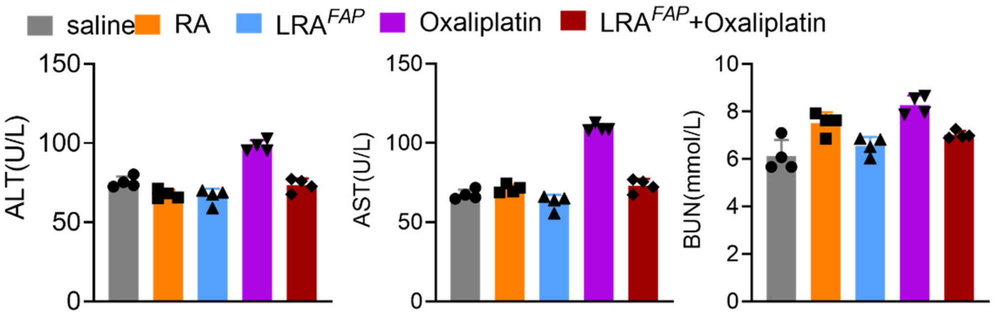

Supplementary Figure S1. Synthetic routes for RA–linoleyl alcohol and oncoFAP–linoleyl alcohol conjugates.

(A) Synthesis of the retinoic acid–linoleyl alcohol conjugate (RA–LA) via EDC/DMAP-mediated esterification in anhydrous dichloromethane (DCM). (B) Synthesis of the oncoFAP–linoleyl alcohol conjugate (oncoFAP–LA) via EDC/DMAP-mediated esterification in anhydrous N, N-dimethylformamide (DMF).

Supplementary Figure S2 | Validation of a TGF-β–induced CAF-like NIH/3T3 model.

(A) qPCR analysis showed that, relative to untreated NIH/3T3 cells, TGF-β treatment significantly increased the

relative expression of Fap and Acta2 (encoding α-SMA), confirming successful induction of a CAF-like phenotype. Statistical significance is indicated by asterisks **$P < 0.01$, ***$P < 0.001$.

Supplementary Figure S3 | Quantitative analysis of tumour accumulation in bilateral MC38 and MC38/CAF tumours. (A) Representative fluorescence images of excised MC38 and MC38/CAF tumours collected after intravenous administration of LRA@DiR or LRA^FAP@DiR. (B) Quantitative analysis of average radiance in MC38 and MC38/CAF tumours following intravenous administration of LRA@DiR or LRA^FAP@DiR **$P < 0.01$.

Supplementary Figure S4 | Percentage change in tumour volume from baseline at study endpoint.

Percentage change in tumour volume from baseline at study endpoint (tumour volume change from baseline, %).

Supplementary Figure S5 | Serum biochemistry indices for hepatic and renal toxicity evaluation. Serum biochemistry indices—alanine aminotransferase (ALT), aspartate aminotransferase (AST) and blood urea nitrogen (BUN)—to evaluate potential hepatic and renal toxicity.
